# Blood‐based Biomarkers for Alzheimer's Disease and Memory Function in Midlife

**DOI:** 10.1002/alz70857_104460

**Published:** 2025-12-25

**Authors:** Alyssa C Kam, Christopher R. Beam, Eric Turkheimer, Ebrahim Zandi, Bruna Martins‐Klein, Sophie A. Bell, Deborah Finkel, Deborah Winders Davis

**Affiliations:** ^1^ University of Southern California, Los Angeles, CA, USA; ^2^ University of Virginia, Charlottesville, VA, USA; ^3^ Jönköping University, Jönköping, Sweden; ^4^ University of Louisville School of Medicine, Louisville, KY, USA; ^5^ Norton Children's Research Institute affiliated with the University of Louisville School of Medicine, Louisville, KY, USA

## Abstract

**Background:**

Clarifying risk factors for Alzheimer's disease (AD) in midlife permits intervening earlier in the lifespan and delaying conversion to AD. Understanding the relationship between blood‐based AD biomarkers and memory functioning during middle age may help clarify whether elevated levels correspond to poor memory performance in later life. Blood‐based biomarkers, including amyloid‐β42/amyloid‐β40 and ptau181, are associated with AD diagnosis, but studies investigating the correlates of these biomarker levels in middle age are scarce (Li et al., 2022; Karikari et al., 2020).

**Method:**

Blood‐based AD biomarkers and episodic memory scores from the California Verbal Learning Test‐ 3^rd^ Edition (CVLT‐3) were collected from 154 midlife twins (78 complete families) as part of the Louisville Twin Study. Pearson correlations and mixed effects regression analysis were used to estimate between‐family and within‐family associations between AD biomarkers and four CVLT‐3 subtests (immediate word recall, short‐delay free recall, long‐delay free recall, and recognition).

**Result:**

Correlations of amyloid‐β42/amyloid‐β40 and the CVLT3 subtests ranged from ‐.02 to .04 while correlations with ptau181 ranged from ‐.09 to ‐.04. Although no correlations were statistically significant, the pattern of associations between episodic memory and ptau181 at least suggested a reliable negative association. Neither between‐family nor within‐family correlations were observed.

**Conclusion:**

Although AD biomarker levels have been found to correlate with memory functioning in older adult samples, we did not observe these same effects in middle adulthood. Thus, the current results suggest that individual differences in blood‐based AD biomarkers may not have predictive utility for observed memory functioning in middle adulthood.

